# Functional Probiotic Assessment and* In Vivo* Cholesterol-Lowering Efficacy of* Weissella* sp. Associated with Arid Lands Living-Hosts

**DOI:** 10.1155/2018/1654151

**Published:** 2018-11-14

**Authors:** Imene Fhoula, Amel Rehaiem, Afef Najjari, Donatella Usai, Abdellatif Boudabous, Leonardo Antonio Sechi, Ouzari Hadda-Imene

**Affiliations:** ^1^Université de Tunis El Manar, Faculté des Science de Tunis, LR03ES03 Laboratoire Microorganismes et Biomolécules Actives, 2092, Campus Universitaire, Tunis, Tunisia; ^2^Università degli Studi di Sassari, Dipartimento di Scienze Biomediche, Sezione di Microbiologia Sperimentale e Clinica, Viale San Pietro 43/B, 07100 Sassari, Italy

## Abstract

The research and the selection of novel probiotic strains from novel niches are receiving increased attention on their proclaimed health benefits to both humans and animals. This study aimed to evaluate the functional properties of* Weissella* strains from arid land living-hosts and to select strains with cholesterol-lowering property* in vitro* and* in vivo*, for use as probiotics. They were assessed for acid and bile tolerance, antibiotic susceptibility, membrane properties, antibacterial activity, antiadhesive effect against pathogens to host cell lines, and cholesterol assimilation* in vitro*. Our results showed that the majority of strains revealed resistance to gastrointestinal conditions. All the strains were nonhemolytic and sensitive to most of the tested antibiotics. They also exhibited high rates of autoaggregation and some of them showed high coaggregation with selected pathogens and high adhesion ability to two different cell lines (Caco-2 and MIM/PPk). Particularly,* W. halotolerans *F99, from camel feces, presented a broad antibacterial spectrum against pathogens, reduced* Enterococcus faecalis *and* Escherichia coli* adhesion to Caco-2 cells, and was found to reduce,* in vitro*, the cholesterol level by 49 %. Moreover,* W. halotolerans* F99 was evaluated for the carbohydrate utilization as well as the serum lipid metabolism effect in Wistar rats fed a high-cholesterol diet.* W. halotolerans *F99 showed an interesting growth on different plant-derivative oligosaccharides as sole carbon sources. Compared with rats fed a high-fat (HF) diet without* Weissella* administration, total serum cholesterol, low-density lipoprotein cholesterol, and triglycerides levels were significantly (*p*<0.001) reduced in* W. halotolerans* F99-treated HF rats, with no significant change in high-density lipoprotein cholesterol HDL-C levels. On the basis of these results, this is the first study to report that* W. halotolerans *F99, from camel feces, can be developed as cholesterol-reducing probiotic strain. Further studies may reveal their potential and possible biotechnological and probiotic applications.

## 1. Introduction

Probiotics are defined as “live microorganisms, which, when administrated in adequate numbers, confer a health benefit to the host” [1]. Lactic acid bacteria (LAB), especially Lactobacilli, are widely used in food production and represent the most common microorganisms employed as probiotics in functional foods [2–4]. The probiotic concept is gaining much worldwide attention, due to the perceived beneficial effects of these bacteria on human and animal health [2, 5, 6]. As well, the use of probiotics has been rising in order to ovoid negative effect induced by the abusive use of antibiotics in human and veterinary medicine [7, 8]. Particularly, the use of antibiotic as growth promoters in animal feed has been suspected to be responsible for the emergence of multidrug-resistant pathogens [9–11]. Therefore, the possibility of using probiotics as preventive/curative treatment or human and animal health promoters constitutes an important subject in applied microbiology [12, 13]. Probiotic strain selection must satisfy many criteria related to their safety, persistence, and the required functional characteristics [14]. The tolerance to gastric acid and bile toxicity, the adhesion ability to intestinal cells, and inhibition of pathogenic bacteria are among the most important probiotic properties to consider in candidates selection for gastrointestinal tract colonization [2, 14]. Probiotic bacteria have their origins mainly from fermented foods or the gut microflora of humans and animals. However, based on many clinical studies the efficacy of some probiotics is highly questionable, such as* Lactobacillus rhamnosus* GG strain causing sepsis in children and adult patients linked to their ingestion as probiotic supplements [15–18]. Thus, the rigorous characterization and evaluation of the probiotic abilities are the most important factors for probiotic candidates. Particularly, bacterial species have not been before reported as probiotics. Moreover, the isolation and the selection of novel probiotic strains from other ecological niches could have the advantage to obtain strains with new beneficial functional properties, potentially useful for technological and/or probiotic applications. Organisms living in arid lands represent a valuable source to prospect for the selection of potential probiotic bacteria. The difference of origin should lead to specific bacterial characteristics, which might provide a new or prominent probiotic effect to the patients. Such organisms may select specific microorganisms having particular metabolic traits in response to their adaptation to hard conditions [19]. This concept of the possible implication of gut microbiome of many living organisms, especially for insects, in the survival/ adaptation of their hosts, becomes noticeable and well-argued [19–21]. In this work, LAB from desert plants and gastrointestinal microflora of camels and* Cataglyphis* ants were studied and evaluated for their probiotic potential. In fact, camels and* Cataglyphis* ants have a high capacity of adaptation to survive in semiarid, arid, and desert areas, which are characterized by poor nutrients, high temperatures, salt stress, desiccation, and UV radiations [20]. In addition,* Cataglyphis* ants are one of the most distinctive groups of insects that live in arid regions. They are commonly considered a model organism for studying many advanced adaptation traits [20]. Moreover, most ant species and their larvae are edible in different parts of the world, in order to satisfy the growing demand for sustainable feed and food sources [22]. Therefore, these distinctive physiological characteristics gained by such desert host-organisms may emphasize the presence of a peculiar gut microbiota, endowed with interesting metabolic properties contributing to the adaptation of their hosts.* Weissella* was proposed as a relatively new genus among the members of LAB, based on 16s rRNA gene sequences phylogeny data [23]. It is among the most widespread lactic species in different ecological niches [24]. Bacteria of the genus* Weissella* encompassing 19 species are reported to be isolated from a variety of fermented plant-based foods, soil, plants, animal products, human feces, and the gastrointestinal tract of human or animal [24–26]. They are facultative anaerobes and commonly grow at temperatures between 15 and 42°C. Only few studies have reported the evaluation of* W. kimchii, W. confusa*, and* W. cibaria* strains as potential probiotics [26, 27]. The aim of this study was to evaluate the probiotic prospective of some selected* Weissella* strains, isolated from unexploited source (bacterial communities associated with camel feces, gut of* Cataglyphis* ants, and desert plants), and to assess,* in vivo* the cholesterol-lowering effect of selected probiotic potential* Weissella* strains.

## 2. Materials and Methods

### 2.1. Sampling Methods

Samples of desert plants (*Euphorbia guyoniana*) (13), camel feces (49), and ants (15) were collected from arid land of southern Tunisia in March 2010 and 2011. The different samples were collected in sterile bags autoclaved or falcon tubes (Thermo Scientific Nunc, 50 ml), kept below 10°C, and treated within 7 days. LAB from plant samples were isolated by the enrichment method as described by Fhoula et al. [28]. LAB isolation from feces was performed as described by Foo et al. [29]. The* Cataglyphis* ants were transferred in sterile containers for organ dissection before microbial isolation. The ants were surface-disinfected with 70% ethanol and rinsed twice with sterilized water prior to dissection. Each adult ant was aseptically dissected using sterilized fine-tip forceps and the entire gut was removed from the body. Guts were placed in 1.5 ml tubes with 500 *μ*l of physiological saline (0.85% NaCl). After that, they were macerated with a plastic pestle and used for bacteria culturing.

### 2.2. Bacterial Strains, Culture Conditions, and Cell Lines

From a total of 69 environmental LAB isolates of the* Weissella *genus, nine strains were retained for this study based on a preliminary selection of resistance to low pH (see below), one of the more important selection criteria for probiotics (Table 1)*. Enterococcus faecium *MMRA [30] was associated with this study. LAB were cultured in De Man, Rogosa, and Sharpe MRS broth (Biolife) at 37°C. Other bacterial strains used for antibacterial activity, including* Escherichia coli* DH5*α*,* Listeria monocytogenes* L15,* Salmonella *Typhimurium IPT13,* Enterococcus faecalis* ATCC 29212,* Pseudomonas aeruginosa* ATCC 27853, and* Staphylococcus aureus* ATCC 6538, were grown in BHI broth (Biolife) at 37°C. Identification of the isolated strains was performed as described by Fhoula et al. [28] using 16S rRNA gene sequence analysis. The obtained DNA sequences were deposited in the GenBank database and the corresponding accession numbers are indicated in Table 1. For long-term storage, the strains were stored at −80°C in 15% glycerol. The human Caucasian colon adenocarcinoma Caco-2 cell line was purchased from* Sigma*-Aldrich. The Murine enteric glial MIM/PPk cell line [31] was provided as a gift by Prof. Anne Ruehl (University of Munich). The opportunistic pathogens (*Escherichia coli* N176 and* Enterococcus faecalis* P592) used for the inhibition adhesion to Caco-2 cells experiment were obtained from the collection of Biomedical Sciences Department, Section Experimental and Clinical Microbiology, University of Sassari, Italy. All chemicals required for cell culturing and adhesion studies were purchased from Sigma-Aldrich, USA. The Caco-2 cells were grown in RPMI 1640 medium supplemented with 10% (v/v) foetal bovine serum (FBS, Sigma) and 100 U/ml penicillin-streptomycin (Sigma-Aldrich). The MIM/PPk cells were cultured in DMEM-F12 medium (Dulbecco Minimal Essential Medium, GIBCO) supplemented with 10% FBS and 100 U/ml penicillin-streptomycin. Incubation was made at 37°C in the presence of 5% CO_2_. Cells were seeded at a concentration of 1x10^5^ cells per well on coverslips inside 24-well tissue culture plates.

### 2.3. Tolerance to Low pH and Bile

The tolerance of* Weissella* strains to low pH was tested as described by Klayraung et al. [32]. Acid resistance in MRS broth adjusted to pH 2.5 with 1N HCl for 90 min at 37°C was used as preliminary screening for probiotic strain evaluation. The ability of* Weissella* strains to resist this pH was determined by single streaking on MRS agar plates after 48 h of anaerobic incubation at 37°C. Tolerance of the selected strains to pH 2.2 was conducted as follows: cell pellets were washed twice in 0.01M phosphate buffered saline (PBS, 0.14 M NaCl, 1.5 mM K_2_HPO_4_, 6.0 mM Na_2_HPO_4_, 3.0 mM KCl; pH 7.4) and resuspended in 10 ml of (0.05M) phosphate buffer pH 2.2 (adjusted using 1N HCl) to achieve 10^7^-10^8^ CFU/ml and were held at 37°C for 2 h. Cells were serially diluted 10-fold in phosphate buffer (0.1 M, pH 6.2). To test the resistance to bile salts,* Weissella* strains were grown in MRS broth containing 0.3 and 0.5% (w/v) of bile salts for 3 h incubation at 37°C. The viable bacterial cells under acidic and bile conditions were determined by plating in triplicate on MRS agar after an incubation of 48 h at 37°C. The survival rate was calculated as the percentage of colonies growing on MRS agar, compared with the initial bacterial concentration.

### 2.4. Adhesion Assays to Caco-2 and MIM/PPk Line Cells

Adhesion ability of LAB strains to intestinal epithelial cells of the enterocyte-like Caco-2 cell line and to MIM/ PPk murine intestinal glial cells was investigated. Briefly, LAB strains were grown in BHI-GY medium for 18 h at 35°C. The cells were harvested (10000 x g, 10 min, RT), washed twice with sterile PBS, diluted in DMEM, and adjusted to 0.5 McFarland. Cell monolayers were washed with antibiotic-free DMEM and 1 ml of bacterial suspension (approximately 1x10^6^ CFU/ml) was added to each well. Plates were centrifuged at 1500 x g for 10 min. After incubation for 3 h at 37°C with 5% CO_2_, the plates were washed two times with PBS and fixed with methanol for 30 min. After staining with May-Gruenwald/ Giemsa solution (Riedel-de-Haёn, Germany), bacterial adherence to the cells was visualized by light microscope (Zeiss optical microscope), under oil immersion, at a magnification of 100x [33]. Two independent experiments were performed for each strain and uninfected cells were included as a negative control. Adherent LAB in 20 random microscopic fields (40 in total for each strain) were counted.

### 2.5. Autoaggregation and Coaggregation

The autoaggregation and coaggregation assays for* Weissella* strains were determined according to Malik et al. [34]. The coaggregation capacity of* Weissella *isolates was examined with respect to the tested bacterial partner strains of* E. coli *DH5*α, S. *Typhimurium IPT13, and* St. aureus* ATCC 6538. The autoaggregation and coaggregation percentages were determined as the percent decrease of optical density (OD_660_) of the nonaggregated cells in the supernatant after 60 min using the following equation: Aggregation % = [(OD_t0_ − OD_t60_)/OD_t0_] x 100.

### 2.6. Hemolytic Activity and Antibiotic Resistance

Fresh bacterial cultures were streaked in triplicate on base blood agar plates with 5% (v/v) horse blood and incubated at 30°C for 48 h. Blood agar plates were checked for *β*-haemolysis, *α*-haemolysis, or *γ*-haemolysis [35]. Susceptibility to antibiotics was determined by using the disk diffusion method on Muller Hinton agar (MHA) plates supplemented with 0.2% glucose and 0.4% yeast extract. The antibiotics used were ampicillin (AM; 10 *μ*g), chloramphenicol (C; 30 *μ*g), erythromycin (E; 15 *μ*g), tetracycline (TE; 30 *μ*g), clindamycin (CL; 2 *μ*g), rifampicin (RA; 5 *μ*g), and vancomycin (VAN; 30 *μ*g) (Bio-Rad Laboratories, Hercules, CA, USA). MH plates were overlaid with soft MHA (containing 0.7% agar) inoculated at 0.5 McFarland with fresh bacterial culture. After 24 h of incubation at 37°C, the inhibition zone diameters around discs were measured, and the LAB isolates were categorized, according to the standard criteria [36], as resistant (R), intermediate resistant (I), or sensitive (S).

### 2.7. Inhibition of Pathogenic Bacteria

The inhibitory activity of* Weissella* strains against the indicator used strains was assayed by the agar spot test described by Schillinger and Lücke [37] with some modifications. Spots of 3 *μ*l of each LAB culture were deposited onto the surface of LBP agar plates [tryptone (20 g), yeast extract (5 g), lactose (10 g), gelatin (2.5 g), agar (11 g), NaCl (0.4 g), sodium acetate (1.5 g), and distilled water (1L)]. Then, they were incubated at 35°C for 24 h to allow the colonies to develop. The indicator strains (*St. aureus*,* L. monocytogenes*,* En. faecalis*,* S. *Typhimurium,* P. aeruginosa*, and* E. coli*) were inoculated into 5 ml of soft agar (0.7% agar) at the concentration of 10^5^-10^6^ CFU/ml and poured over the plate on which the LAB isolates were grown. After incubation at 35°C for 24 h, the plates were examined for the presence of clear inhibition zones. Inhibition was considered positive when the diameter of the clear zone around the spot of the LAB isolates was more than 5 mm. All antibacterial tests were performed in triplicate.

### 2.8. Inhibition of Pathogen Adhesion to Epithelial Cells Caco-2

For exclusion assays of pathogen bacteria from adhering to Caco-2 cells, 100 *μ*l of* Weissella* bacterial suspension (ca. 1× 10^8^ CFU) was added to Caco-2 cells in each well, as described above, and incubated for 90 min at 37°C. Monolayers of Caco-2 cells were washed twice with 1 ml of sterile PBS to release unbound bacteria and then inoculated with 100 *μ*l (10^8^ CFU/ml) of one of the following opportunistic gastrointestinal and urogenital pathogens:* E. coli *N176 and* En. faecalis* P592 (resistant to Beta-lactamin, glycopeptides, penicillin, and vancomycin). After incubation, unbound bacterial cells were removed from wells and the Caco-2 cells were washed twice with 1 ml of sterile PBS, followed by 1 ml of 0.5% (v/v) Triton X-100 to release adhering bacterial cells. Serial dilutions were plated on MRS agar (Biolife), MacConkey agar (MCA, Biolife), and Bile Esculin agar (BEA, Biolife) media to enumerate* Weissella *species,* E. coli*, and* En. faecalis,* respectively. For competition assays, the competitive inhibition of the pathogens by the tested* Weissella* strains was determined as described previously, except that LAB strain and one of the pathogens (*E. coli* and* En. faecalis*) were added simultaneously to the Caco-2 cultures and incubated for 3 h at 37°C. Wells containing pathogenic bacteria alone served as controls. The capacity of selected* Weissella* strains to exclude or to inhibit the adhesion to Caco-2 cells from potential gastrointestinal pathogens was expressed as a percentage between the adhesion of pathogens in the presence and in the absence of the tested* Weissella* strain.

### 2.9. Phenotype Microarrays

The growth on different carbon sources (93) of two* Weissella* strains was assessed using Phenotype Microarray (PM) Technology (Biolog, Hayward, CA). Bacterial cells from a single colony, grown on BHI agar for 48 h, were suspended in the specific Biolog medium (adjusted to 65% of transmittance) and used to inoculate the phenotype microarray 96-well plates (PM1 and PM2), according to the manufacturer's instructions. PM plates were incubated for 72 h at 37°C. Data from a single experiment were analyzed with Omnilog-PM software. For each carbon source, the metabolic activity was measured quantitatively based on the area under curve. The two independent replicates of each PM plate showed the same results.

### 2.10. Cholesterol Assimilation

*Weissella* cells were inoculated into sterile MRS broth containing 0.3% (w/v) oxgall (Sigma) and 100 *μ*g/ml filter sterilized water-soluble cholesterol (Sigma) and incubated anaerobically at 37°C for 24 h. Cells were harvested and the residual cholesterol concentration in the supernatant was determined using the o-phthalaldehyde colorimetric method of Rudel and Morris [38]. The percentage of cholesterol removed by the strain compared to the control was calculated as follows: [1- (residual cholesterol in cell-free broth)/cholesterol of control broth)] x 100.

### 2.11. *In Vivo* Cholesterol Assays

#### 2.11.1. Animals and Experimental Design

Adult male Wistar rats, weighing 165.1 ± 5.2 g, were purchased from Pasteur Institute of Tunisia and housed two per clean plastic cages and allowed to acclimatize in the laboratory environment. The animal room was ventilated and maintained with 12 h light/dark at 24°C and a relative humidity of 50%. The rats were provided standard diet and water ad libitum. Animal experiments were carried out under strict compliance with the Guidelines for Ethical Control and Supervision in the Care and Use of Animals. After acclimatization, a total of 16 animals were randomly selected and divided into two groups (n=8 for each one). Groups I and II were fed with high-fat HF containing diet for 2 weeks and then treated as follows: group I received HF diet with PBS (control group), and group II received HF diet and* W. halotolerans* in PBS, 9×10^9^ CFU/Kg body weight of suspension* W. halotolerans* F99 in PBS. A sterile gastric feeding tube was used for orally inoculating one of the two groups daily with 1 ml of* W. halotolerans* F99 suspension during 8 weeks at 9×10^9^ CFU/Kg body weight. At the end of the experimental period, rats were sacrificed by decapitation in order to minimize the handling stress, and the trunk blood was collected. The serum was prepared by centrifugation (2500 ×* g*, 10 min, 4°C), frozen, and stored at −20°C until it was analyzed for the plasma lipid profile. The HF diet contained 1% wt/wt cholesterol, 10 % oil fat, and a normal diet mix.

#### 2.11.2. Serum Lipids

The concentrations of total cholesterol (TC), high-density lipoprotein cholesterol (HDL-C), and triglycerides (TG) in serum were determined by enzymatic colorimetric methods using commercial kits (Elitech, France), while the low-density lipoprotein cholesterol (LDL-C) was calculated according to Friedewald's formula [39].

### 2.12. Statistical Analysis

Statistical analysis was done through SPSS 19.0 software (SPSS Inc, Chicago, IL, USA). Data obtained were analysed using one-way analysis of variance (ANOVA) and Tukey's test. Data were considered significantly different at* p*-value less than 0.05. All data are expressed as the mean ± standard deviation.

## 3. Results and Discussion

### 3.1. Tolerance to Low pH

Acid tolerance constitutes one of the first criteria used to select probiotic microorganisms for their ability to survive transit through the stomach [40]. The potential probiotic LAB strains isolated from different sources were first evaluated to survive to low pH (pH 2.5) condition. Tolerance to pH (2.2) was also checked for the selected strains. The results revealed that most LAB strains could survive approximately less than 68% up to 89.3% under low pH (Table 1) for 2 hours, which is the average time required for a classic passage of the food in the stomach [41]. The most tolerant strains were* W. halotolerans* FAS23, FAS24, and F99 with a survival rate ranging between 72.1 and 89.2 %. Particularly,* W. halotolerans* F99 survived better than probiotic* En. faecium* MMRA from dairy product (85.4%). Besides*, W. halotolerans *FAS65 was sensitive to this pH value at survival rate of 44.5% (Table 1). These results suggest that the resistance to low pH is a strain dependent property and the gut origin ecosystem could play an essential role for the bacteria to be able to adapt to the stress environments.

### 3.2. Bile Tolerance

Tolerance of LAB cells to different concentrations of bile salts (0.3% and 0.5%) in MRS was evaluated. The most strains, except* W. halotolerans* FAS22, showed a significant survival rate after 3 h of growth in physiological (0.3%) and high concentrations (0.5%) of bile (Table 1). But overall, survival rates were lower compared to positive control at 0.3% bile. Taking into consideration the acidity criterion,* W. halotolerans *FAS3,* W. halotolerans* F99, and* W. confusa* F80 were found among the resistant strains to 0.3% of bile, reaching a viability rate up to 73%. Besides, we noted an increase in the number of viable cells of some LAB in the high concentration 0.5% of bile (Table 1). These results highlight the potential of some* Weissella* isolates to survive under gastrointestinal conditions. Indeed, the high tolerance to bile salts represents an important factor that may considerably influence the viability of LAB in the host gastrointestinal tract and for the exploitation of these strains as probiotics. Hence, it is a prerequisite for the colonization and the contribution of metabolic activity of bacteria in balancing the intestinal microflora of their host [42]. Based on the gastrointestinal tolerance assays, five strains (F80, F99, FAS23, FAS3, and FAS24) were selected with survival rate of over 68% for further evaluation of other probiotic properties.

### 3.3. Adhesion to Caco-2 and MIM/PPk Cell Lines

**Figure 1:**
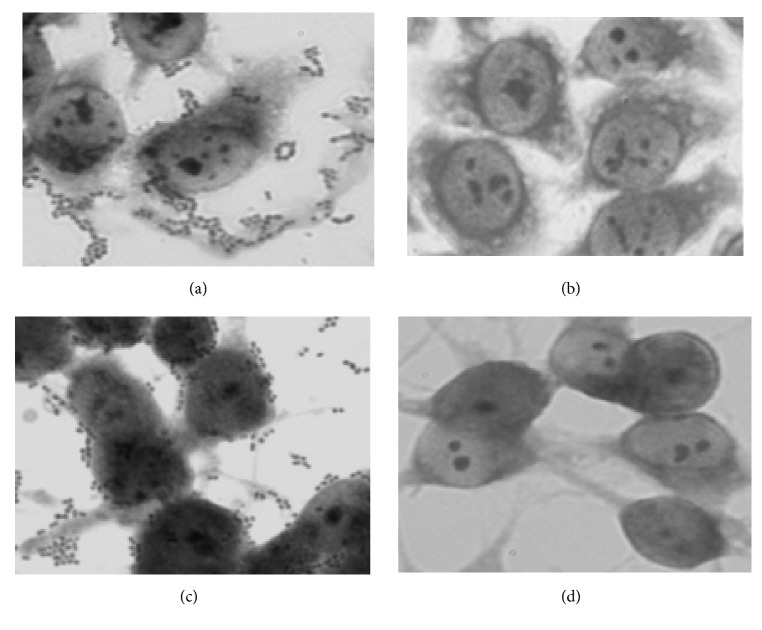
**Adhesion of* Weissella* strains to Caco-2 epithelial (a) and MIM/PPK (b) enteric glial cells, as observed with Giemsa staining under a light microscope (magnification X 100).** (c) and (d), recognized as the cells without bacterial adhesion as a negative control.

The adhesion ability of* Weissella* strains was studied for two types of cell lines (Figure 1): the human colon carcinoma cell line (Caco-2), as an excellent* in vitro* enterocyte model, and the enteric glial cells (MIM/PPk), a major constituent of the enteric nervous system that appears to be essential for the maintenance of gut homeostasis and mucosal integrity [43–45]. Indeed, the enteric glial cells are known to play a complex and fundamental role in regulating many neuronal activities and seem to be involved in immunological and inflammatory processes in the gut [44, 45]. The adherence ability of* Weissella* toward the cell lines was different. They were able to adhere well or strongly to at least one of the two tested cell lines (Table 2). Particularly* W. confusa* F80 has presented strong specific adhesion only for MIM/PPk. The variable adhesion ability to different cell lines may reflect the mode of action of these bacteria [46]. Therefore, the strong adhesion of bacteria to Caco-2 line cells may facilitate the host colonization and the competitive exclusion of pathogenic bacteria from the epithelium surface. This is the case of strains* W. halotolerans* F99, FAS24, and FAS3, which could be selected as potential probiotic candidates. Considering this data, we could establish a correlation between biofilm formation on abiotic surface and the adherence to Caco-2 cells, except for the* W. halotolerans* FAS23. While the high adhesion to the enteric glial cells (EGC) may indicate that these strains could be involved in immune system modulation [45]. The mechanism of interaction of bacteria to EGCs is currently unknown [44]. In accordance with the suggestion of Ortua et al. [46], the use of combinations of potentially probiotic strains revealing different adhesion abilities may result in complementary effects, which could be exploited for different applications.

### 3.4. Autoaggregation and Coaggregation

Compared to the probiotic* En. faecium* MMRA strain, almost all strains showed a good autoaggregation percentage with an average of 64% (Table 3). The highest value of autoaggregation was observed for* W. confusa* F80 with an aggregation percentage up to 72% after incubation at room temperature for 1 h. Moreover,* W. halotolerans* FAS23, FAS3, and F99 exhibited a good autoaggregating phenotype with a percentage of 66.5%, 64.6%, and 64.1%, respectively. These results indicated that the majority of the tested strains possessed high potential ability to adhere to epithelial cells and mucosal surfaces. In fact, this ability of autoaggregation was related to cell adherence properties [47], which is essential to be effective in the gut flora. On the other hand, the coaggregation of* Weissella* strains with three enteropathogens,* E. coli DH5α, S. *Typhimurium IPT13,and* St. aureus *ATCC 6538, was checked (Table 3). According to Kang et al. [48], the coaggregation percentage was significant when it reduced the level of enteropathogenic bacterial aggregation more than 30%. Excepting* W. halotolerans* FAS24 which demonstrated a weak coaggregation with* E. coli* (10.4%) and* St. aureus* (15.6%), most of* Weissella* strains showed an interesting coaggregation percentage with the tested enteropathogenic bacteria. This ability was particularly registered with* S.* Typhimurium, followed by* E. coli*, and* St. aureus.* The coaggregation abilities of probiotic strains with pathogens play an important role, enabling it to form a barrier that prevents colonization of harmful enteric pathogens usually involved in infectious disease [48, 49]. Likewise, it is also showed by Kang et al. [48] that the coaggregation abilities of some* W. cibaria* isolates with the oral biofilm-forming pathogen* Fusobacterium nucleatum* play an important host defence mechanism against infection by their interference against the biofilm formation.

### 3.5. Safety Evaluation of* Weissella* Strains: Hemolysin and Antibiotic Susceptibility

Hemolytic test and resistance to some antibiotics were checked for the studied* Weissella* strains in order to evaluate their safety and to avoid their contribution to virulence. The results showed that the selected-*Weissella* strains were nonhemolytic and presented some resistance phenotypes (Table 4). Indeed, all strains were sensitive to chloramphenicol, clindamycin, and ampicillin, but also they had intrinsic resistance to vancomycin, which does not present potential risk for horizontal gene transfer [50].* W. halotolerans* (FAS23 and FAS3) and* W. confusa* F80 were discarded from further studies on the basis of their acquired resistance to tetracycline (Table 4). In fact, the use of drug-resistant and/or virulent bacteria as probiotics represents a potential health hazard. For that reason, the safety evaluation of probiotics is required to avoid risks related to antibiotic and virulence gene transfer and dissemination, which contribute to the pathogenesis of virulent bacteria [51]. Moreover, additional tests of toxicity, pathogenicity, and infectivity should be performed in order to establish the “safety” status of the selected strains [52]. On the basis of these results, two potential probiotic strains of* W. halotolerans* (F99 and FAS24) were selected for further study.

### 3.6. Antibacterial Activity against Intestinal Pathogens

As shown in Table 5, the antagonistic effect of the two selected* Weissella* strains against six pathogenic bacteria was greatly variable. Contrary to* W. halotolerans* FAS24,* W. halotolerans* F99 presented a broad spectrum of antibacterial activity against both Gram-positive and Gram-negative enteropathogenic bacteria. Particularly, it showed high inhibition activity against invasive* S.* Typhimurium and* P. aeruginosa *isolates, which were reported to penetrate epithelial cell monolayers and to cause intestinal infections [53, 54]. The isolate* W. halotolerans *FAS24 showed only an antibacterial inhibition of* St. aureus*. The different antibacterial responses observed in* Weissella* strains against various pathogenic bacteria indicated that these activities could not relate only to acidity. Indeed, the inhibitory activity of LAB is generally due to its ability to produce antibacterial molecules such as lactic acid, bacteriocins, H_2_O_2_, and other organic acids [55]. Besides, the antibacterial property detected in* W. halotolerans *F99 led to suggestion that it enables the bacteria to establish themselves and to dominate their environment.

### 3.7. Inhibition of the Adhesion of Pathogens to Caco-2 Cells by Two Weissella Strains

**Figure 2:**
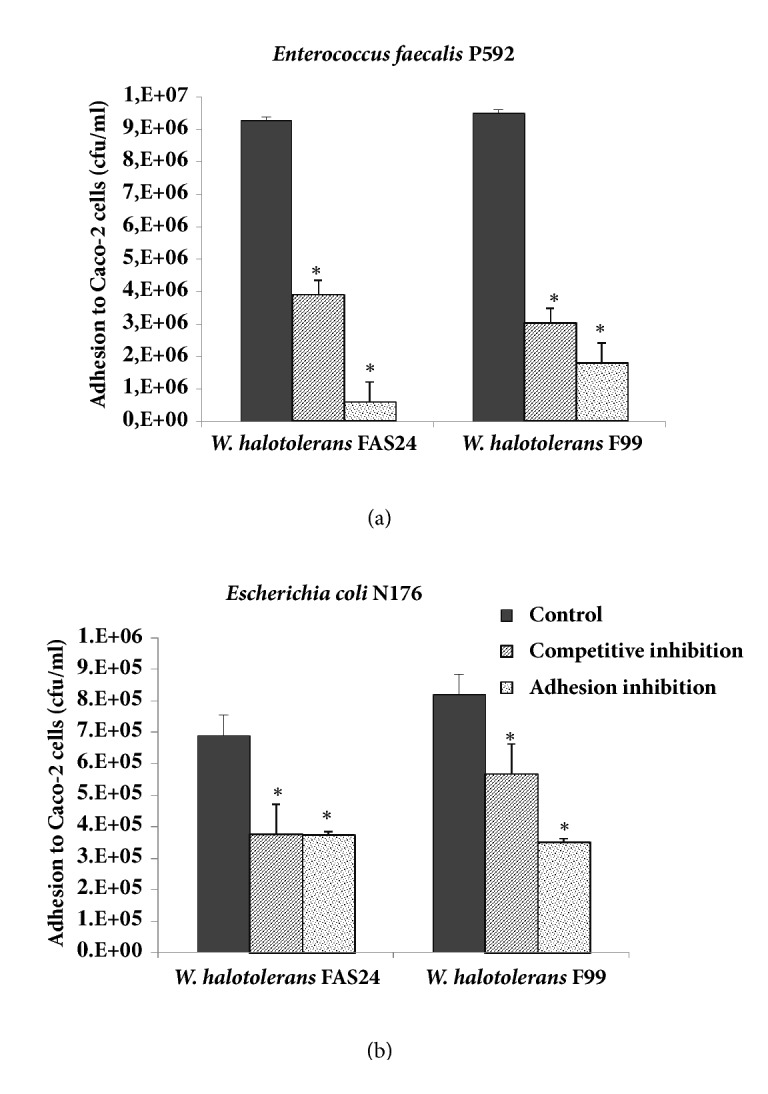
**Competitive and exclusion inhibition of adhesion of* Enterococcus faecalis *(a) and* Escherichia coli *(b) to the Caco-2 cells by* Weissella halotolerans* (F99 and FAS24) strains.** Asterisks indicate significant differences (*∗ p* < 0.001).

We examined the effect of two* Weissella* strains on the antiadhesion activity against* En. faecalis *P592 and* E. coli* N176 to Caco-2 cells in two conditions of competition and exclusion assays (Figure 2). Adhesion of the pathogens was inhibited by both* Weissella* strains. For competitive inhibition, the adhesion of* En. faecalis* was considerably reduced by* W. halotolerans* F99 (68%) and* W. halotolerans* FAS24 (58%) compared to* E. coli* (Figure 2). On the other hand, the two* Weissella* strains were tested for their ability to exclude pathogens. As shown in Figure 2,* W. halotolerans* FAS24 and* W. halotolerans* F99 significantly reduced the adhesion of* En. faecalis* with a high degree of exclusion of 94% and 81%, respectively, whereas they showed a moderate inhibition of enteropathogenic* E. coli* with an average of 50%. Surprisingly, the competition and exclusion inhibition profiles of* En*.* faecalis* by the two* Weissella* strains were nearly similar. This confirms that these two adhesion inhibition mechanisms of* En. faecalis* by these LAB strains are similar. Besides, our results suggest that the ability to reduce the pathogen adhesion was strain-dependent in both the LAB and the pathogen tested. This fact may be due to different factors such as the steric hindrance of available adhesion sites, adhesin receptors, and competition for attachment sites and to other factors such as coaggregation of both strains [56–58]. Similarly, the specific adhesion system to Caco-2 cell lines, which appears to be different between Gram-positive and Gram-negative bacteria, as well as the absence of antibacterial activity against* E. coli* could explain the moderate inhibitory activity of adhesion recorded with* E. coli* cells compared to* En. faecalis. *As revealed above, these findings suggest that the production of inhibitory substances can participate efficiently in the antiadhesive effect of the pathogen to epithelial cells [59].

### 3.8. Carbon Source Utilization by Phenotype Microarrays

The carbohydrate utilization profile of two selected strains (*W. halotolerans* F99 and FAS24) was investigated using Phenotype Microarray (Biolog) in order to determinate the metabolic functions of probiotic interest (Table S1 in file S1). Carbon source utilization was different between the tested strains (Table S1 in file S1). These strains were generally able to use glucosamine, gluconic acid, ribose, inosine, aminoethanol, dextrin, arabinose, and arbutin.* W. halotolerans *F99 was able to metabolize mannitol, xylose, arabinose, ribose, maltose, gentiobiose, glucoside, and tween. Particularly,* W. halotolerans* F99 displayed an important growth rate on plant-derivative complex carbohydrates such as xylose, cellobiose, trehalose, gentiobiose, and galactose [60]. Most of the metabolized oligosaccharides and particularly those containing arabinose and xylose substituents (commonly called arabinoxylan oligosaccharides or AXOS) cannot be degraded by human enzymes of the GIT. Probiotic fecal microbes are indispensable for the degradation of these molecules. This activity is responsible for the formation of partial carbohydrate breakdown products and short chain fatty acids, leading to the maintenance of a balanced gut homeostasis [61]. Moreover, such oligosaccharides are commonly used as prebiotic that stimulate the activity of specific probiotic bacteria of the colon and increase their abundance.

### 3.9. Cholesterol In Vitro and In Vivo Assays

The elevated serum cholesterol level is considered a risk factor of cardiovascular disease [62]. Therefore, cholesterol assimilation has become an important functional property for selection of probiotic strains to prevent disease. The cholesterol-reducing ability of LAB strains was evaluated* in vitro* in the presence of 0.3% bile. The results indicated that* W. halotolerans* F99 and FAS24 strains had the ability to remove cholesterol from the medium; however, they exhibited varying ratios of cholesterol-lowering ability of 49.04±0.04% and 19.63±0.10%, respectively. The assimilation rate* exhibited by W. halotolerans* F99 was comparable to the probiotics* Lb. rhamnosus* GG and* Lb. plantarum* NR74 showing an average of 48% [11] and higher than some probiotic reference* Lactobacillus* spp. reported in previous studies [63].

**Figure 3:**
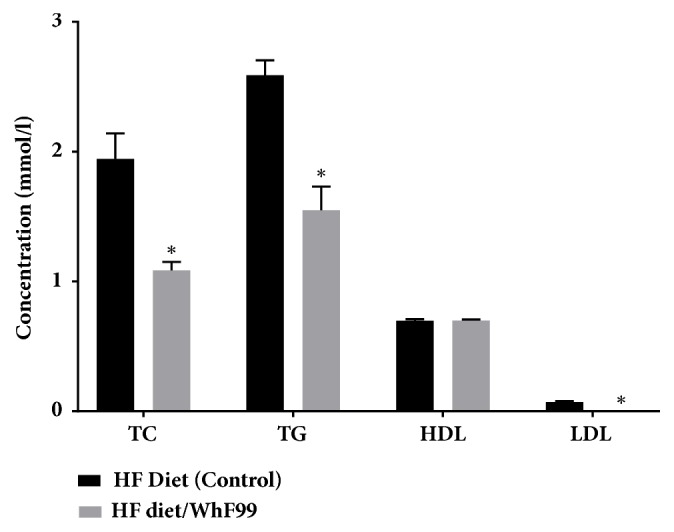
**Serum lipid levels of the control and treated groups after 8 weeks.** Control group: high-fat diet; treated group: high-fat diet+* W. halotolerans* F99 (WhF99). Each concentration is the mean ± standard deviation (n = 8). Asterisks indicate significant differences (*∗ p* < 0.001 vs. control).

Owing to its high cholesterol assimilation ability,* W. halotolerans *F99 was assessed for the* in vivo* effect in Wistar rats. The administration of this* Weissella* strain to rats fed with high-fat diet was found to affect their serum lipid profile (Figure 3). Compared with the control group, the values for total cholesterol of rats serum (TC), triglyceride (TG), and LDL were reduced significantly (p<0.001) in group fed with* W. halotolerans *F99. However, for HDL this difference was not statistically significant (Figure 3). Similar results were also reported by Nocianitri [64] and Bendali [65] showing the effectiveness of probiotics to improve lipid profile* in vitro *and* in vivo*. These data provided for the first time the screening of* Weissella* strains for their cholesterol reduction ability. These results represent a preliminary basis for the promising role of* W. halotolerans *F99 as probiotic-based therapies, which may be used for the treatment and prevention of cholesterol metabolism and metabolic diseases, such as development of functional food with probiotic supplement.

## 4. Conclusions

In the view of our data, the* in vitro* assessment of probiotic properties of* Weissella* strains from arid land living-hosts was shown to be strain specific. The majority of the tested strains have showed to possess interesting probiotic features, including resistance to gastrointestinal conditions, cell surface properties, and adhesive ability to Caco-2 and MIM/PPk cells, as well as the inhibition and the competitive exclusion of harmful pathogens. The present study led to the first-line selection of* W. halotolerans* F99, from camel feces, as a putative strain for future studies as it was found to fit the almost required probiotic properties, including adhesion to epithelial cells, carbohydrate utilization, and cholesterol-lowering effect. It could be used as potential probiotic adjunct to improve the lipid profile in animal and human health. However, the mechanism(s) of regulating serum cholesterol needs further investigations by such a promising probiotic candidate. Based on the findings of this study the gut microflora of camel serves as a special source for model strains.

## Figures and Tables

**Table 1:** Origin and survival rate (%) of selected *Weissella *strains to low pH and different bile concentrations.

**Strains**	**Source**	**Survival rate (**%**) at pH2.2**	**Survival rate (**%**) in bile after 3 h**	
			**0.3**%	**0.5**%
*W. cibaria *V28 (KM100709)	Plant (*Euphorbia guyoniana*)	61.63±0.20	64.46±0.01	82.9±0.03
*W. confusa *F81 (KM100707)	Camel feces	53.28±0.13	76.26±0.10	50.96±0.01
*W. confusa *F80 (KM100708)	Camel feces	68.89±0.17	77.80±0.03	85.16±0.01
*W. halotolerans *F99 (KM100706)	Camel feces	74.24±0.12	78.78±0.03	96.28±0.05
*W. halotolerans *FAS23 (KM100711)	Ant gut	89.29±0.03	72.00±0.25	54.65±0.02
*W. halotolerans* FAS22 KM100710)	Ant gut	60.20±0.09	29.86±0.34	-
*W. halotolerans* FAS3 (KM100705)	Ant gut	69.97±0.18	73.96±0.05	56.33±0.02
*W. halotolerans* FAS65 (KF198087)	Ant gut	44.45±0.14	75.33±0.05	50.23±0.09
*W. halotolerans* FAS24 (KF198085)	Ant gut	72.10±0.11	68.00±0.80	42.60±1.10
*En. faecium *MMRA	Dairy product	85.42±0.01	89.14±0.03	75.60±0.02

W, *Weissella*; *En*., *Enterococcus*. Each value represents the mean value ±standard deviation (SD) from three trials. (-), No growth; (*∗*), Survival rate of bacterial cells successively treated in a low pH and high bile.

**Table 2:** Adherence of cells of *Weissella *strains to Caco-2 and MIM/PPk cell lines.

**Strains**	**A** **d** **h** **e** **s** **i** **o** **n** ^ǂ^	
	**Caco-2**	**MIM/PPk**
*W. confusa* F80	NA	+++
*W. halotolerans* F99	+++	++
W. *halotolerans* FAS23	++	++
W. *halotolerans* FAS3	+++	+++
W. *halotolerans* FAS24	+++	+++

(*ǂ*), NA: No significant adhesion (< 40); (+), weak adhesion, 40 ≤ Nb < 200); (++), Good adhesion (200 ≤ Nb <1000); (+++), strong adhesion (≥1000). Each value represents the mean value ±standard deviation (SD) from three trials. Adherence was evaluated in 20 random microscopic fields.

**Table 3:** Percentage of autoaggregation and coaggregation of five selected *Weissella* strains.

**Strains**	**Autoaggregation** **(**%**± SD)**	**Coaggregation (**%**± SD) with**		
		***Escherichia coli DH5*** **α**	***Salmonella *Typhimurium* IPT13***	***Staphylococcus aureus ATCC 6538***
*W. confusa *F80	72.27±0.55	68.26±0.35	69.24±0.27	59.65±1.18
*W. halotolerans* F99	64.12±1.81	68.33±1.36	71.70±0.98	50.90±0.91
*W. halotolerans *FAS23	66.51±1.12	74.00±0.63	81.13±0.11	75.38±0.33
*W. halotolerans* FAS3	64.61±0.39	80.61±0.49	79.25±0.17	67.08±1.35
*W. halotolerans* FAS24	52.10±0.64	10.40±1.06	78.81±0.07	15.64±0.23
*En. faecium *MMRA	54.20±0.42	21.10±1.81	68.35±0.67	46.12±1.53

Each value represents the mean value standard deviation (SD) from three trials. Values are significantly different (*P <* 0.05).

**Table 4:** Antibiotic susceptibility, hemolytic activity of selected *Weissella* strains.

**Strains**	**Vancomycin**	**Erythromycin**	**Chloramphenicol**	**Tetracycline**	**Clindamycin **	**Ampicillin**	**Rifampicin**	**Hemolytic**
	VA (30*μ*g)	E (15*μ*g)	CH (30*μ*g)	TE (30*μ*g)	CL (15*μ*g)	AM (10*μ*g)	RA (5*μ*g)	Activity
*W. confusa *F80	6±0 (R)	36±2 (S)	24±1 (S)	14±0 (R)	34±2 (S)	27±2 (S)	27±1 (S)	*γ*-hemolytic
*W. halotolerans *F99	6±0 (R)	43±0 (S)	30±1 (S)	20±1 (S)	38±2 (S)	23±1 (S)	33±2 (S)	*γ*-hemolytic
*W. halotolerans *FAS23	6±0 (R)	25±0 (S)	22±1 (S)	14±0 (R)	29±1 (S)	25±1 (S)	27±1 (S)	*γ*-hemolytic
*W. halotolerans *FAS3	6±0 (R)	35±2 (S)	25±1 (S)	14±0 (R)	18±0 (S)	23±1 (S)	20±0 (S)	*γ*-hemolytic
*W. halotolerans* FAS24	6±0 (R)	37±1 (S)	31±1 (S)	22±0.5 (S)	30±0 (S)	23±0 (S)	31±0 (S)	*γ*-hemolytic

S, sensitive; R, resistant; The numbers represent the diameter of zone of inhibition (mm).

**Table 5:** The antibacterial activity of the selected *Weissella* strains against six pathogenic bacteria.

**Strains**	***Escherichia coli DH5α***	***Salmonella *Typhimurium* IPT13***	***Staphylococcus aureus ATCC 6538***	***Listeria monocytogenes LM15***	***Pseudomonas aeruginosa ATCC 27853***	***Enterococcus faecalis ATCC 29212***
*W. halotolerans *F99	-	21±0.4	12±1	12.3±1.5	24.1±0.6	12.4±0.3
*W. halotolerans *FAS24	-	-	12±1.6	-	-	-

Numbers indicated the diameter of the inhibition zone in mm; each value represents the mean value standard deviation (SD) from three trials.

## Data Availability

The nucleotide sequences data used to support the findings of this study are publicly available in the GenBank repository at National Center for Biotechnology Information NCBI (https://www.ncbi.nlm.nih.gov/genbank/). All data are provided in full in Results and Discussion in this paper. The results of Biolog phenotypic microarray analysis data used to support the findings of this study are included within the supplementary information file.

## Supplementary Materials

Supplementary Materials Supplementary Table S1: positive reaction for carbohydrate utilization by two* Weissella halotolerans* strains using Biolog phenotypic microarray.
