# Supplementary material for: Functional Probiotic Assessment and In Vivo Cholesterol-Lowering Efficacy of Weissella sp. Associated with Arid Lands Living-Hosts
Source: Biomed Res Int. 2018 Nov 14;2018:1654151. doi: 10.1155/2018/1654151 (PMC6261067; doi:10.1155/2018/1654151)
Supplement: Supplementary Materials — Supplementary Table S1: positive reaction for carbohydrate utilization by two Weissella halotolerans strains using Biolog phenotypic microarray. [file 1654151.f1.doc]

**Supplemental material**

**Supplementary Table S1:** Positive reaction for carbohydrate utilization by two Weissella halotolerans strains using Biolog phenotypic microarray.

| **Substrats** | ***W. halotolerans* F99** | ***W. halotolerans* FAS24** |
| --- | --- | --- |
| **L-Arabinose** | +++ | - |
| **N-Acetyl-D- Glucosamine** | +++ | ++ |
| **D-Galactose** | +++ | - |
| **D-Trehalose** | +++ | - |
| **D-Mannose** | +++ | + |
| **Dulcitol** | - | +++ |
| **Glycerol** | - | +++ |
| **D-Gluconic Acid** | +++ | +++ |
| **D-Xylose** | ++ | - |
| **D-Mannitol** | +++ | - |
| **D-Ribose** | +++ | ++ |
| **Tween 20** | +++ | - |
| **D-Fructose** | +++ | - |
| **Alpha-D-Glucose** | +++ | - |
| **Maltose** | +++ | - |
| **Thymidine** | +++ | - |
| **Tween 40** | +++ | - |
| **Alpha-D-Lactose** | - | - |
| **Uridine** | +++ | ++ |
| **Tween 80** | +++ | - |
| **Maltotriose** | - | ++ |
| **Adenosine** | ++ | - |
| **D-Cellobiose** | ++ | - |
| **Inosine** | ++ | + |
| **N-Acetyl-beta-D- Mannosamine** | - | ++ |
| **L-Lyxose** | - | +++ |
| **2-Aminoethanol** | + | ++ |
| **Alpha-Cyclodextrin** | + | - |
| **Beta-Cyclodextrin** | + | - |
| **Gama-Cyclodextrin** | + | - |
| **Dextrin** | + | ++ |
| **N-Acetyl-D- Galactosamine** | - | - |
| **D-Arabinose** | +++ | + |
| **Arbutin** | + | +++ |
| **2-Deoxy-D- Ribose** | +++ | +++ |
| **Gentiobiose** | +++ | - |
| **L-Glucose** | - | - |
| **a-Methyl-D- Glucoside** | +++ | - |

(-), absence of activity; (+), weak activity; (++), moderate activity; (+++), strong activity.
